# Disruption of the expression and function of microRNAs in lung cancer as a result of epigenetic changes

**DOI:** 10.3389/fgene.2013.00275

**Published:** 2013-12-03

**Authors:** Kousuke Watanabe, Daiya Takai

**Affiliations:** ^1^Department of Respiratory Medicine, The University of Tokyo HospitalBunkyo-ku, Tokyo, Japan; ^2^Department of Clinical Laboratory, The University of Tokyo HospitalBunkyo-ku, Tokyo, Japan

**Keywords:** microRNA, lung cancer, DNA methylation, histone modification, RNA editing

## Abstract

Two decades have passed since the discovery of microRNA (miRNA), which determines cell fate in nematodes. About one decade ago, the conservation of miRNA in humans was also discovered. At present, the loss of certain miRNAs and the overexpression of miRNAs have been demonstrated in many types of diseases, especially cancer. A key miRNA in lung cancer was reported soon after the initial discovery of a tumor-suppressive miRNA in a hematological malignancy. Various causes of miRNA disruption are known, including deletions, mutations, and epigenetic suppression as well as coding genes. The recent accumulation of knowledge regarding epigenetic transcriptional suppression has revealed the suppression of several miRNAs in lung cancer in response to epigenetic changes, such as H3K9 methylation prior to DNA methylation and H3K27 methylation independent of DNA methylation. In this review, recent knowledge of miRNA disruption in lung cancer as a result of epigenetic changes is discussed. Additionally, emerging cancer-specific changes in RNA editing and their impact on miRNA function are described.

## MICRORNAs AND CANCER: A HISTORICAL PERSPECTIVE

MicroRNAs (miRNAs) are small non-coding RNA molecules (approximately 22 nucleotides) that function as versatile regulators of gene expression. miRNAs negatively regulate the expression of thousands of genes through the destabilization and/or translational suppression of mRNAs by binding to complementary sequences in the 3′ untranslated regions (3′UTRs) of target mRNAs (Lee et al., 1993; Wightman et al., 1993).

The first miRNA to be discovered, *lin-4*, was determined to be an essential regulator of development in the nematode *Caenorhabditis elegans* (Lee et al., 1993; Wightman et al., 1993). This short non-coding RNA was considered to be a peculiar constituent specific to worms. Seven years passed before a second miRNA, *let-7*, was discovered in nematodes (Reinhart et al., 2000). Shortly thereafter, *let-7* was found to be broadly conserved across many species, including humans (Pasquinelli et al., 2000). In 2001, a large number of such genes were identified, and the term “microRNA” was coined (Lagos-Quintana et al., 2001; Lau et al., 2001; Lee and Ambros, 2001). Currently, more than 2,000 mature miRNAs have been documented in the miRNA registry (Sanger miRBase release 20; ).

MicroRNAs are involved in many biological processes such as cell cycle control, cell differentiation, and apoptosis. Alterations in miRNA expression have been increasingly recognized as playing important roles in the pathogenesis of human cancers. For example, the first tumor-suppressive miRNAs *mir-15* and *mir-16* located at 13q14 are frequently deleted and downregulated in chronic lymphocytic leukemia (Calin et al., 2002). In lung cancer, a reduction in *let-7* expression is significantly associated with a shorter postoperative survival (Takamizawa et al., 2004). The *let-7* miRNAs target important oncogenes such as the *Ras* family (Johnson et al., 2005) and *HMGA2* (Mayr et al., 2007). The *mir-17-92* miRNA cluster, which was the first oncogenic miRNA to be reported, is amplified and over expressed in B cell lymphoma (He et al., 2005). Moreover, the *mir-17-92* miRNA cluster is also amplified and overexpressed in small-cell lung cancer (SCLC) and enhances the proliferation of cancer cells (Hayashita et al., 2005).

MicroRNAs can be used as biomarkers for the diagnosis and prognosis of malignancies. In general, miRNA expression is downregulated in tumors, compared with normal tissues, and analyzes of the expression of 217 miRNAs in various human cancers reflect the developmental lineage and differentiation of the tumor (Lu et al., 2005). Furthermore, certain miRNAs can aid in classifying the histological subtype (adenocarcinoma or squamous cell carcinoma) of lung cancer (Bishop et al., 2010). The miRNA signature can also predict the survival and relapse of patients with lung cancer (Yu et al., 2008).

Despite growing evidence of the involvement of miRNAs in human carcinogenesis, limited information is available regarding how miRNA expressions are deregulated in cancer. In this article, we review the mechanisms responsible for the changes in miRNA expression in lung cancer, focusing particularly on epigenetic mechanisms, such as DNA methylation and histone modifications.

## MECHANISMS OF DEREGULATED miRNA EXPRESSION IN CANCER

**FIGURE 1:**
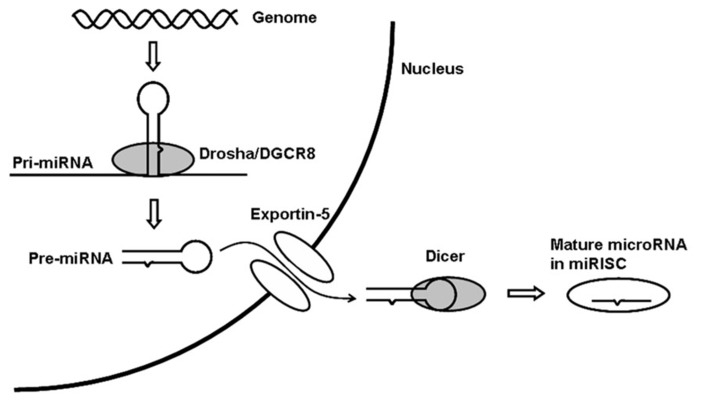
**miRNA biogenesis pathway. ** miRISC, microRNA-induced silencing complex.

In animals, miRNAs are generally transcribed by RNA polymerase II (Lee et al., 2004) to form primary transcripts (pri-miRNAs). Pri-miRNAs form hairpin structures in the nucleus and are processed by the Drosha/DGCR8 complex to form approximately 60 nt precursor miRNAs (pre-miRNAs; Gregory et al., 2004). Pre-miRNAs are transported to the cytoplasm through the RAN GTP-dependent transporter exportin-5 (Lund et al., 2004) and are cleaved by Dicer into mature miRNAs (Hutvagner et al., 2001; **Figure 1**).

miRNA are frequently located at fragile sites as well as minimal regions of loss of heterozygosity, minimal regions of amplification, or common breakpoint regions in cancer (Calin et al., 2004). In addition to such genomic changes, any alteration in the miRNA biogenesis pathway described above can affect miRNA expression in cancer. The currently known mechanisms responsible for changes in miRNA expression in cancer include genomic deletions or amplifications, chromosomal translocations, epigenetic silencing by DNA methylation, and impairments of the miRNA biogenesis pathway, such as the frameshift mutation of *exportin-5* (Melo et al., 2010), the downregulation of Dicer (Karube et al., 2005; Merritt et al., 2008), and the frameshift mutation of *TARBP2* (Melo et al., 2009).

As mentioned above, *mir-15* and *mir-16*, located at 13q14, are deleted in more than half of all cases of chronic lymphocytic leukemia (Calin et al., 2002), and amplification of the *mir-17-92* miRNA cluster located at 13q31 is observed in B cell lymphoma and SCLC (Hayashita et al., 2005; He et al., 2005). In addition, *mir-125b-*1 is a target of the chromosomal translocation *t*(11,14) in B-cell precursor acute lymphoblastic leukemia, and this translocation results in *mir-125b* overexpression that is controlled by an immunoglobulin heavy-chain gene regulatory element (Bousquet et al., 2008; Chapiro et al., 2010). However, with regard to lung cancer, no chromosomal translocations involving miRNAs have been previously reported.

Kumar et al. (2007) reported that the impairment of the miRNA biogenesis pathway in cancer results in the global downregulation of miRNA and the enhancement of cellular transformation and tumorigenesis. They demonstrated that the knockdown of DGCR8, Drosha, or Dicer in cancer cells resulted in a pronounced transformed phenotype and that the conditional deletion of *Dicer* enhanced tumor development in a *K-ras*-induced mouse model of lung cancer. Importantly, a reduction in Dicer expression is associated with a poor prognosis in patients with lung cancer (Karube et al., 2005) and ovarian cancer (Merritt et al., 2008). Interestingly, a germline mutation of *Dicer1* has been discovered in pleuropulmonary blastoma, a rare pediatric lung tumor (Hill et al., 2009). These findings clearly demonstrate that the miRNA biogenesis pathway plays a crucial role in normal lung development and lung carcinogenesis. Frameshift mutations of *exportin-5* and *TARBP2* have been reported in colorectal cancer with microsatellite instability, but not in lung cancer (Melo et al., 2009, 2010).

Epigenetic changes in cancer genomes, such as DNA methylation and histone modifications, cause the silencing of tumor suppressor genes and contribute to human carcinogenesis (Jones and Takai, 2001; Jones and Baylin, 2002; Herman and Baylin, 2003). Recently, DNA methylation in cancerous tissue has been shown to silence miRNAs in cancer (Saito et al., 2006; Lujambio et al., 2007). Saito et al. reported that the expression of *mir-127*, which is embedded in a CpG island, was induced by treatment with the chromatin-modifying drugs 5-aza-2’-deoxycytidine and 4-phenylbutyric acid in a bladder cancer cell line. Lujambio et al. analyzed the miRNA expression profile of a colon cancer cell line, which was genetically deficient for the DNA methyltransferase (DNMT) enzymes *DNMT1* and *DNMT3b*, and identified the epigenetic silencing of *mir-124a* in various types of cancer, including colon, breast, and lung cancers as well as leukemia and lymphoma. As the epigenetic silencing of key tumor suppressor genes, such as *APC* and *p16INK4A*, is a common event in lung carcinogenesis (Takai et al., 2001; Sano et al., 2007; Brock et al., 2008; Kusakabe et al., 2010) and miRNA expression is altered in lung cancer (Yanaihara et al., 2006), some miRNAs are thought to be silenced by DNA methylation or histone modification in lung cancer. In fact, growing evidence demonstrates that epigenetic changes contribute to miRNA silencing in lung cancer (Liu et al., 2013).

## DNA METHYLATION AND miRNA EXPRESSION

The earliest papers on miRNA expression profiling in lung cancer were published in 2006 (Volinia et al., 2006; Yanaihara et al., 2006). These studies used miRNA microarrays and found that a high level of *mir-155* expression and a low level of *let-7a-2* expression were significantly correlated with a poor survival of lung cancer patients. The number of miRNA profiling studies in lung cancer has grown rapidly in recent years, and these studies have led to the discovery of the role of miRNAs in lung carcinogenesis and their potential as diagnostic, prognostic, or predictive markers (Rothschild, 2013). Vosa et al. performed a meta-analysis of 20 published miRNA expression profiling studies in lung cancer and identified a meta-signature of seven up-regulated (*mir-21, mir-210, mir-182, mir-31, mir-200b, mir-205, and mir-183*) and eight down-regulated (*mir-126-3p, mir-30a, mir-30d, mir-486-5p, mir-451a, mir-126-5p, mir-143, and mir-145*) miRNAs (Vosa et al., 2013). Guan et al. (2012) also reported a meta-analysis of 14 published miRNA expression profiling studies and their results agreed well with those published by Vosa et al.

One approach to identifying epigenetically silenced miRNAs is to analyze the miRNA expression profile of cancer cells before and after treatment with chromatin-modifying drugs. Lujambio et al. analyzed the miRNA expression profile of three metastatic cancer cell lines with and without the DNMT inhibitor 5-aza-2’-deoxycytidine and reported that the DNA methylation of three miRNAs (*mir-9*, *mir-34b/c,* and *mir-148a*) was associated with the metastasis of human cancers including lung cancer (Lujambio et al., 2008). Heller et al. analyzed the miRNA expression profile changes in A549 lung cancer cells treated with 5-aza-2’-deoxycytidine and the histone deacetylase inhibitor tricostatin A and identified *mir-9-3* and *mir-193a* as targets for DNA methylation in non-small cell lung cancer (NSCLC; Heller et al., 2012). Our research team analyzed the expressions of 55 *in silico* selected candidate miRNAs treated with or without 5-aza-2’-deoxycytidine and found that *mir-34b/c* and *mir-126* are silenced by DNA methylation in NSCLC (Watanabe et al., 2012). We also revealed that the DNA methylation of *mir-9-*3, *-124-2*, and *-124-3* was individually associated with an advanced T factor, and that the methylation of multiple miRNA loci was associated with a poor prognosis (Kitano et al., 2011). The correlation between miRNA methylation and the T factor suggested that the DNA methylation of these miRNA loci accumulates during tumor progression. A list of miRNAs that are silenced by DNA methylation in lung cancer is shown in **Table 1**.

**Table 1:** miRNAs silenced by DNA methylation in lung cancer.

miRNA	Target genes	Reference
mir-9-3		Lujambio etal. (2008), Kitano etal. (2011), and Heller etal. (2012)
mir-34a, -34b/c	Bcl-2, Cyclin D1, Cyclin E2, CDK4, CDK6, c-Myc, c-Met	Lodygin etal. (2008), Lujambio etal. (2008), Gallardo etal. (2009), Wang etal. (2011), Tanaka etal. (2012), and Watanabe etal. (2012)
mir-124-1, -124-2, -124-3	CDK6	Lujambio etal. (2007) and Kitano etal. (2011)
mir-126	Crk, VEGF-A	Watanabe etal. (2012)
mir-148a	TGIF2	Lujambio etal. (2008) and Chen etal. (2013)
mir-193a		Heller etal. (2012)
mir-200, -205	ZEB1, ZEB2	Tellez etal. (2011)
mir-487b	SUZ12, BMI1, WNT5A, MYC, K-ras	Xi etal. (2013)

The *mir-34* family is comprised of three miRNAs (*mir-34a*, *mir-34b*, and *mir-34c*) that are derived from two transcripts (*mir-34a* on chromosome 1 and *mir-34b/c* on chromosome 11). In mice, *mir-34a* is ubiquitously expressed, with the highest expression being in the brain, whereas *mir-34b/c* is mainly expressed in the lung (Bommer et al., 2007). The *mir-34* genes induce cell cycle arrest, cellular senescence, and apoptosis when ectopically expressed (Bommer et al., 2007; He et al., 2007; Welch et al., 2007) through the downregulation of multiple target genes such as *Bcl-2*, *Cyclin D1*, *Cyclin E2*, *CDK4*, *CDK6*, *c-Myc, *and* c-Met* (Hermeking, 2010). Moreover, *mir-34*s have been identified as direct targets of the p53 transcription factor (Bommer et al., 2007; Corney et al., 2007; He et al., 2007), and their expression is induced in response to DNA damage or oncogenic stress. These results indicate that *mir-34*s play a critical role in the tumor-suppressive program governed by p53. Interestingly, the chromosomal locus 1p36, where *mir-34*a is located, has been proposed to harbor a tumor suppressor gene, since a homozygous deletion at this locus has been reported in neuroblastoma, and *mir-34*a has been identified as a candidate tumor suppressor at this locus (Cole et al., 2008).

In lung cancer, *mir-34a* and *mir-34b/c* are targets of epigenetic silencing by DNA methylation (Lodygin et al., 2008; Gallardo et al., 2009; Wang et al., 2011; Watanabe et al., 2012). In primary NSCLC, a low *mir-34a* expression level is significantly associated with a high probability of relapse after surgery (Gallardo et al., 2009). We previously reported that *mir-34b/c* is methylated in 41% of primary NSCLC cases and that *mir-34b/c* methylation is associated with lymphatic invasion (Watanabe et al., 2012). The DNA methylation of *mir-34b/c* is associated with a poorer prognosis in patients with NSCLC (Wang et al., 2011). In addition, the *mir-34*s are silenced by DNA methylation in SCLC (Tanaka et al., 2012). In primary SCLC, *mir-34a* and *mir-34b/c* were methylated in 15% and 67% of the cases, respectively. The CpG island methylation of *mir-34b/c* has also been reported in colorectal cancer (Toyota et al., 2008), oral squamous cell cancer (Kozaki et al., 2008), melanoma, and breast cancer (Lujambio et al., 2008). Thus, the epigenetic inactivation of *mi-34*s is a common event in human cancer.

*mir-34a* and *mir-34b/c* are intergenic miRNAs, and their expressions are regulated by the DNA methylation of their own promoters. Importantly, many miRNA encoding sequences are located within the introns of protein coding genes, and intronic miRNAs are usually expressed in a coordinate manner along with their host gene mRNAs (Baskerville and Bartel, 2005). We previously reported that *mir-126*, which is located within the intron of *EGFL7*, is silenced by the DNA methylation of its host gene in NSCLC (Watanabe et al., 2012). *mir-126* functions as a tumor-suppressive miRNA, suppressing metastasis in breast cancer (Tavazoie et al., 2008) and inhibiting the invasion of NSCLC cell lines by targeting *Crk* (Crawford et al., 2008). Moreover, *mir-126* was recently reported to be an essential regulator of angiogenesis. *Vascular endothelial growth factor (VEGF)-A* is a target of *mir-126*, and the downregulation of *mir-126* increases the activity of VEGF-A in lung cancer (Liu et al., 2009). A reduction in *mir-126* expression is significantly associated with increased microvessel density in oral squamous cell cancer (Sasahira et al., 2012) and NSCLC (Jusufovic et al., 2012), suggesting a negative regulatory role of *mir-126* in tumor angiogenesis. In addition, decreased *mir-126* expression is significantly associated with a shorter survival period in patients with NSCLC (Jusufovic et al., 2012). In contrast, Donnem et al. demonstrated that an elevated level of *mir-126* expression is associated with a shorter survival period in patients with NSCLC and that an elevated level of *mir-126* expression was associated with an increase in *VEGF-A* expression in NSCLC (Donnem et al., 2011). The targeted deletion of *mir-126* in mice impaired normal angiogenesis, suggesting a positive regulatory role of *mir-126* in angiogenesis (Wang et al., 2008). Additional research is required to elucidate the relation between *mir-126*, tumor angiogenesis, and tumor progression. Furthermore, we reported that the DNA methylation of *EGFL7* (a host gene of *mir-126*) was only observed in 7% of the clinical samples that were evaluated (Watanabe et al., 2012), which cannot completely explain the frequent downregulation of this miRNA in NSCLC. In fact, our analyzes of DNA methylation of the coding genes (Sano et al., 2007; Kusakabe et al., 2010) and miRNAs (Kitano et al., 2011; Watanabe et al., 2012) revealed that the ratio of DNA methylation is often quite low in primary NSCLCs. This low ratio of DNA methylation may be a result of insufficient DNA methylation following changes in histone modification, rather than the result of the coexistence of non-cancerous tissues, and may be responsible for the low frequency of DNA methylation of *mir-126* in primary NSCLCs if the method used to detect DNA methylation is not sufficiently sensitive.

Cigarette smoking is the most important risk factor for lung cancer. Accumulating evidence suggests that tobacco induces the epigenetic silencing of certain miRNAs in lung carcinogenesis (Tellez et al., 2011; Xi et al., 2013). Tellez et al. reported that the exposure of human bronchial epithelial cells (HBECs) to tobacco carcinogens decreased the expressions of *mir-200b*, *-200c*, and *-205* and induced the epithelial-to-mesenchymal transition (EMT). The *mir-200* family and *mir-205* function as key negative regulators of the EMT through the direct targeting of *ZEB1* and *ZEB2* (Gregory et al., 2008; Park et al., 2008). In HBECs, tobacco carcinogens initially induced an increase in H3K27me3 (inactive closed chromatin) and subsequently induced the DNA methylation of sequences encoding these miRNAs. The loss of *mir-200c* expression as a result of DNA methylation has been shown to induce an aggressive, invasive, and chemoresistant phenotype of NSCLC (Ceppi et al., 2010). In addition, Shien et al. (2013) demonstrated that a lung cancer cell line with acquired resistance to an EGFR tyrosine kinase inhibitor exhibited EMT features and the downregulation of *mir-200c* as a result of DNA methylation. Xi et al. reported that tobacco carcinogens induced the epigenetic silencing of *mir-487b* and that *mir-487b* functions as a tumor suppressive miRNA in NSCLC by targeting *SUZ12*, *BMI1*, *WNT5A*, *MYC,* and *K-ras*. These studies highlight the potential of epigenetic drugs to reverse tobacco-induced reprogramming in lung cancer cells.

## H3K27me3 AND miRNAs

Epigenetic silencing in mammalian cells is mediated by at least two distinct histone modifications: histone H3 trimethylation at lysine 27 (H3K27me3) and histone H3 dimethylation and trimethylation at lysine 9 (H3K9me2 and H3K9me3). A recent genome-wide study of histone modifications in prostate cancer cells revealed H3K27me3 as a mechanism of tumor-suppressor gene silencing in cancer that occurs independently of promoter DNA methylation (Kondo et al., 2008). A polycomb group protein, EZH2, exhibits histone methyltransferase activity with substrate specificity for H3K27 (Cao and Zhang, 2004). EZH2 overexpression is associated with a poor prognosis in lung cancer, and the knockdown of EZH2 expression decreases the growth and invasion of lung cancer cells (Huqun et al., 2012). These findings suggest that aberrant H3K27me3 contributes to tumor-suppressor gene silencing in lung cancer, but genome-wide analyzes of H3K27me3 in lung cancer have not been reported.

Recently, Au et al. (2012) analyzed the changes in miRNA expression profiles induced by EZH2 knockdown and found that some tumor-suppressive miRNAs (*mir-139*, *-125b*, *-101*, -*200b*, and *let-7c*) are silenced by H3K27me3 in hepatocellular carcinoma. In lung cancer, *mir-212* is silenced by histone modifications rather than DNA methylation (Incoronato et al., 2011). Incoronato et al. showed that increases in H3K27me3 and H3K9me2 are observed in the *mir-212* promoter region in the lung cancer cell line Calu-1, which has a low *mir-212* expression level. *EZH2* may exert its oncogenic function, at least in part, by silencing tumor-suppressive miRNAs, and further investigation is required to verify the association between H3K27me3 and miRNA expression in lung cancer.

## miRNAs THAT TARGET EPIGENETIC MACHINERY

While miRNA expression is regulated by DNA methylation and histone modifications, genes encoding the epigenetic machinery are also targeted by miRNAs. The *mir-29* family is the prototype of such miRNAs (Fabbri et al., 2007). The mir-29 family is comprised of three miRNAs (*mir-29a*, *mir-29b*, and *mir-29c*) that are derived from two transcripts (*mir-29b-1/29a* on chromosome 7 and *mir-29b-2/29c* on chromosome 1). The *mir-29* family is highly expressed in normal tissues and is downregulated in many types of human cancers including lung cancer (Yanaihara et al., 2006; Xu et al., 2009). *mir-29a* reportedly functions as an anti-metastatic and anti-proliferative miRNA in lung cancer (Muniyappa et al., 2009). *mir-29b* has also been reported to function as an anti-metastatic miRNA in lung cancer through the regulation of the *Src*-*ID1 *pathway (Rothschild et al., 2012).

Recently, the *mir-29* family was shown to directly target *DNMT3A* and *DNMT3B*, two enzymes involved in *de novo* DNA methylation (Fabbri et al., 2007). The expression of *mir-29*s is inversely correlated with that of *DNMT3A* and *DNMT3B* in lung cancer tissue, and the enforced expression of *mir-29*s in lung cancer cell lines restores the normal pattern of DNA methylation, induces the re-expression of methylation-silenced tumor suppressor genes (such as *FHIT* and *WWOX*), and inhibits tumorigenicity both *in vitro* and *in vivo*. *mir-29b* also induces *PTEN* expression through the downregulation of DNA methyltransferases (DNMTs) and the subsequent hypomethylation of the *PTEN* promoter in a lung cancer xenograft model (Li et al., 2012). Samakoglu et al. (2012) also report that a combination therapy consisting of an *EGFR* antibody with cisplatin and gemcitabine induces *mir-29b* expression, the downregulation of *DNMT3b*, and the hypomethylation of tumor-suppressor genes in a lung cancer xenograft model. *mir-29b* has also been shown to down-regulate *DNMT1*, an enzyme involved in the maintenance of DNA methylation, indirectly by targeting *Sp1*, a transactivator of the *DNMT1* gene in leukemia (Garzon et al., 2009).

In addition to DNMTs, miRNAs can also target histone modification enzymes. *mir-449a/b* is downregulated and directly targets *HDAC1*, a histone deacetylase in lung cancer (Jeon et al., 2012). *mir-101* reportedly targets *EZH2*, and the genomic loss of *mir-101* leads to the overexpression of *EZH2* in prostate cancer cells (Varambally et al., 2008). These results clearly show a strong interplay between miRNA and the epigenetic machinery, providing new insights into the molecular mechanism of aberrant DNA methylation and histone modifications in cancer.

## RNA EDITING OF miRNAs

The most prevalent type of RNA editing in humans is the deamination of adenosine to inosine in double-stranded RNA (A-to-I editing). This process is catalyzed by two family members of adenosine deaminases acting on RNA (ADAR): ADAR1 and ADAR2. All known A-to-I edited sites have been attributed to *ADAR1* or *ADAR2* (Zinshteyn and Nishikura, 2009). Inosine is present in mRNA at tissue-specific levels that are correlated with ADAR expression. Analyzes of the amount of inosine in various mammalian tissues has revealed that inosine is most abundant in the brain, where one inosine molecule is present for every 17,000 ribonucleotides of mRNA; the second highest frequency of inosine has been observed in the lung, where one inosine molecule is present for every 33,000 ribonucleotides of mRNA (Paul and Bass, 1998).

Recently, certain pri-miRNAs have been reported to be subject to A-to-I editing. Kawahara et al. examined 209 pri-miRNAs and identified 47 pri-miRNAs as the targets of A-to-I editing in the human brain (Kawahara et al., 2008), suggesting that miRNA editing could have a considerable impact on miRNA-mediated gene silencing. Most A-to-I editing of pri-miRNAs results in altered miRNA processing by Drosha and Dicer (Kawahara et al., 2008). However, in rare cases, such as *mir-376*, RNA editing causes base substitution in the seed sequence and generates edited mature miRNAs with unique target genes and functions different from those of the unedited miRNAs (Kawahara et al., 2007).

Emerging lines of evidence suggest a link between A-to-I editing and cancer. Anomalous ADAR activity in cancer may lead to alterations in the efficiency of A-to-I editing. For example, the *glutamate receptor subunit B* (*GluR-B*) is nearly 100% edited at one position (Q/R site) in the normal brain. In primary glioblastoma, this position is substantially under-edited, compared with normal tissues, because of the decreased activity of ADAR2, which is responsible for the A-to-I editing of *GluR-B* (Maas et al., 2001). Recently, the efficiency of A-to-I editing of mir-376a* was found to be significantly attenuated in glioblastoma cells (Choudhury et al., 2012). Unedited mir-376a* promotes the migration and invasion of glioma cells, whereas edited mir-376a* suppresses these features. These results suggest that the attenuation of A-to-I editing of mir-376a* promotes the invasiveness of glioblastoma. Considering the relatively high prevalence of inosine in lung mRNA and the frequent A-to-I editing of miRNAs in the brain, it would be tempting to conduct a large-scale survey to evaluate the A-to-I editing of pri-miRNAs in normal lung and to analyze the alteration of miRNA editing and ADAR activity in lung cancer.

## CONCLUSIONS AND FUTURE PERSPECTIVES

The two major challenges in studying the role of miRNA in cancer are (i) the identification of target genes, and (ii) the elucidation of the mechanisms that regulate miRNA expression in both normal and cancer cells. Limited knowledge is available regarding miRNA transcription, primarily because of inadequate information on the precise locations of the promoters and transcriptional start sites of the miRNAs. Approximately half of all miRNAs are intragenic sequences that are located within the exons, introns, or 3′UTRs of protein-coding genes. These intragenic miRNAs share promoters with their host genes and are co-regulated with their host genes, as in the case of mir-126. The remaining 50% of miRNAs are intergenic miRNAs with their own promoters, which have not been experimentally validated in most cases. Comprehensive analyzes of the miRNA transcription unit will help to elucidate the transcription factors or epigenetic changes responsible for alterations in miRNA expression in cancer.

The impairment of the miRNA biogenesis pathway and the attenuation of A-to-I editing add to the growing complexity of miRNA deregulation in cancer. Moreover, 3′UTRs of certain mRNAs are progressively shortened in cancer cells as a result of changes in alternative cleavage and polyadenylation (APA), a phenomenon that alters the 3′UTR length. Progressive 3′UTR shortening in cancer cells may lead to the disruption of miRNA-mediated gene silencing (Mayr and Bartel, 2009). Understanding these complexities as well as those of miRNA transcriptional regulation may lead to the identification of novel biomarkers and should help to unravel the impact of miRNA in lung carcinogenesis.

## Conflict of Interest Statement

The authors declare that the research was conducted in the absence of any commercial or financial relationships that could be construed as a potential conflict of interest.
